# Pharmacokinetics of a multicomponent herbal preparation in healthy Chinese and African volunteers

**DOI:** 10.1038/srep12961

**Published:** 2015-08-13

**Authors:** Raphael N. Alolga, Yong Fan, Gang Zhang, Jin Li, Yi-Jing Zhao, Jimmy Lelu Kakila, Yan Chen, Ping Li, Lian-Wen Qi

**Affiliations:** 1 State Key Laboratory of Natural Medicines, China Pharmaceutical University, No.24 Tongjia Lane, Nanjing 210009, China; 2 Nanjing Children’s Hospital Affiliated to Nanjing Medical University, No.72, Guangzhou Road, Nanjing 210029, China; 3 Department of Emergency Center, the First Affiliated Hospital of Nanjing Medical University, No. 300 Guangzhou Road, Nanjing 210029, China

## Abstract

K-601 is an herbal formulation for influenza consisting of *Lonicera japonica*, *Isatis indigotica*, *Rheum palmatum*, *Phellodendron chinense*, and *Scutellaria baicalensis*. In this work, we characterized the chemical constituents in K-601, identified the absorbed compounds and determined their pharmacokinetics in 6 Chinese and African volunteers by liquid chromatography with time-of-flight mass spectrometry. Similarity evaluation for chromatographic fingerprint of nine different batches showed values above 0.983. Totally, 50 components were identified in K-601. Then, 15 major prototype compounds and 17 metabolites were identified in human plasma. Major metabolic pathways included glucuronidation, sulfation, methylation, demethylation, and reduction. The pharmacokinetics of the most abundant prototype compounds, berberine, jatrorrhizine, palmatine and magnoflorine were determined. Significant pharmacokinetic differences were observed between the African and Chinese subjects. The *AUC*s of the African is about 4–10 fold higher than that of the Chinese for the three benzylisoquinoline alkaloids. Magnoflorine, an aporphine alkaloid, was absorbed better in the Chinese than in the African. The biotransformation of K-601 by human intestinal microflora was also investigated. The major reactions included hydroxylation, methylation, demethylation, acetylation and reduction. Glucuronidation and sulfation were not observed with fecal flora. These results may be important and useful in linking data from pharmacological assays and clinical effects.

Herbal medicines have gained growing popularity and wide usage in the world in the last twenty years. As estimated by the World Health Organization, 80% of people worldwide rely on herbal medicines for part of their primary health care needs^1^. In China, roughly 1000 herbs are available and are prescribed by TCM practitioners or produced as herbal preparations by pharmaceutical manufacturers^2^. In the United States, an interest in returning to natural or organic remedies has led to an increase in herbal medicines use^3^.

Establishing the evidence-based pharmacokinetics and pharmacodynamics for efficacy of herbal medicines is a constant challenge^4^. Of particular interest is characterization of the complex chemical compositions in herbal medicines. Next of interest are the identification of absorbed compounds and metabolites after oral administration of herbal medicines. Of further interest are the elucidation of metabolic pathways, and the assessment of elimination routes and their kinetics^5^,^6^,^7^.These data become an important issue to link data from pharmacological assays and clinical effects. A better understanding of the pharmacokinetics of phytopharmaceuticals can also help in predicting potential herb-drug interactions and designing rational dosage regimens.

However, pharmacokinetic studies on herbal medicines are very difficult to investigate because of their chemical complexity^8^. As a result, there is very little pharmacokinetic data for many herbal products that are commonly used in clinics and hospitals^9^. In most cases, researchers tend to test the pharmacokinetics of a single compound isolated from an extract using *in vitro* and *in vivo* models^10^. Because of competitive or synergistic absorption and metabolism among various components, differences can be observed between the administration of a single compound and of a compound-contained herbal extract^11^.

A great number of scientists have contributed to developing various methods for analyzing the herbal samples and herbal-treated biological samples^12^,^13^. Advances in sample pretreatment and analytical technology have improved analysis time, sensitivity and efficiency^14^,^15^. An emerging instrument trend has been the application of liquid chromatography combined with mass spectrometry (LC-MS) to online structural characterization and quantification^16^. MS includes quadruple (Q)-MS, ion trap (IT)-MS, time-of-flight (TOF)-MS instruments, and more recently, Q-IT, IT-TOF and Q-TOF^17^,^18^.

K-601 is a hospital-prepared medicinal formulation comprising five herbs, i.e., *Lonicera japonica* Thunb., *Isatis indigotica* Fort., *Rheum palmatum* L., *Phellodendron chinense* Schneid., and *Scutellaria baicalensis* Georgi. It is used in clinic for the alleviation of the symptoms of influenza and treatment of cough due to non-bacterial causes. It is very popular for use in both children and adults in China. Analyses of individual compounds and herbs within this formulation have been studied in terms of chemical composition, quantification, *in vitro* and *in vivo* analysis^19^,^20^. However, no pharmacokinetic studies have been conducted on the unique formulation of K-601 in humans. The aim of this work was to investigate the pharmacokinetics of K-601 in humans by ultra-performance (UP) LC-QTOF/MS, and develop a universal strategy for similar studies of other herbal products. We characterized the chemical constituents in K-601 by a diagnostic-ion screening method, identified the absorbed prototype compounds and their metabolites in healthy Chinese and African volunteers by compound-metabolite matching approach, and determined the pharmacokinetics of some bioactive components. The UPLC-QTOF/MS provided superior data quality and advanced analytical capabilities for profiling, identifying, and determining complex constituents and metabolites in matrix-based biological samples.

## Results

### A strategy proposed to investigate pharmacokinetics of multicomponent herbal preparations

**Figure 1:**
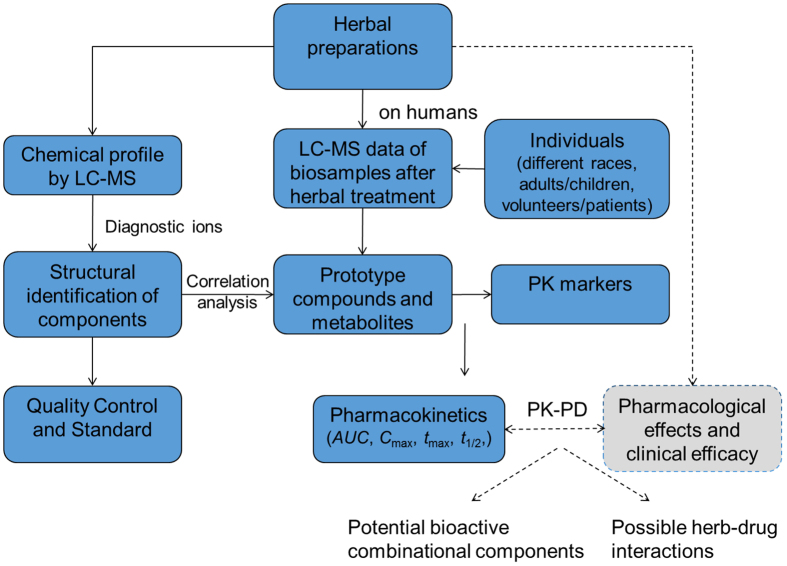
A strategy proposed to investigate pharmacokinetics of multicomponent herbal preparations. → Denotes works done in this work, --> Denotes works will be done in the future.

A strategy was schemed (Fig. 1) to investigate pharmacokinetics of multicomponent herbal preparations. The whole process consists of four steps: (a) chemical profile and structural assignment based on diagnostic ions by LC-MS; (b) LC-MS analysis of biological samples after herbal treatment on humans with different races, ages and volunteers/patients; (c) identification of parent compounds and metabolites in biosamples by data matching; (d) pharmacokinetic studies of high abundant markers in biosamples. Pharmacokinetic studies should be associated with pharmacological effects and clinical efficacy. The results benefit discovery of potential bioactive combinational components and prediction of possible herb-drug interactions.

### Batch to batch quality evaluation of K-601 formulation

**Figure 2:**
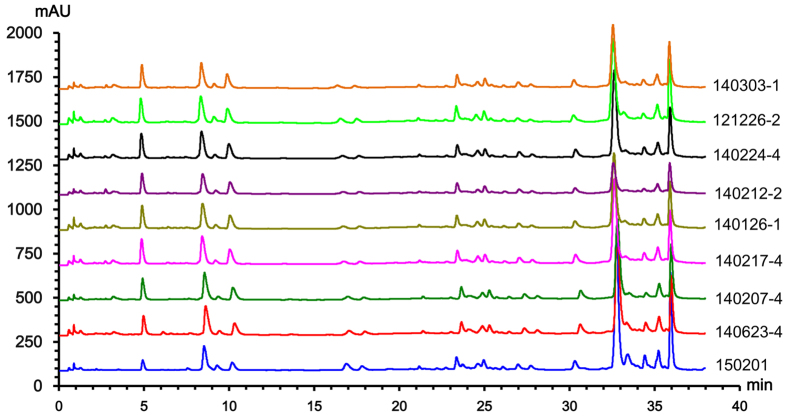
Fingerprint spectra of the nine batches of K-601 used.

To assess the batch to batch consistency of K-601, a simple UPLC method was developed. Fingerprint analysis was conducted on these chromatograms using the software, *Similarity Evaluation System for Chromatographic Fingerprint of TCM, 2004* (Fig. 2). Upon aligning all the peaks, the reference chromatogram was generated by reserving peaks above 0.1% of the percentage area. Peaks that existed in all chromatograms of the samples with reasonable heights and good resolutions were assigned as “common peaks”. The total area of the common peaks must be more than 90% of the peak area of the whole chromatogram. Similarity was reported in terms of cosine ratios (Supplementary Table S1). The results indicated high similarities among the nine batches of K-601 with values higher than 0.983.

### Identification of chemical constituents in formulation

**Figure 3:**
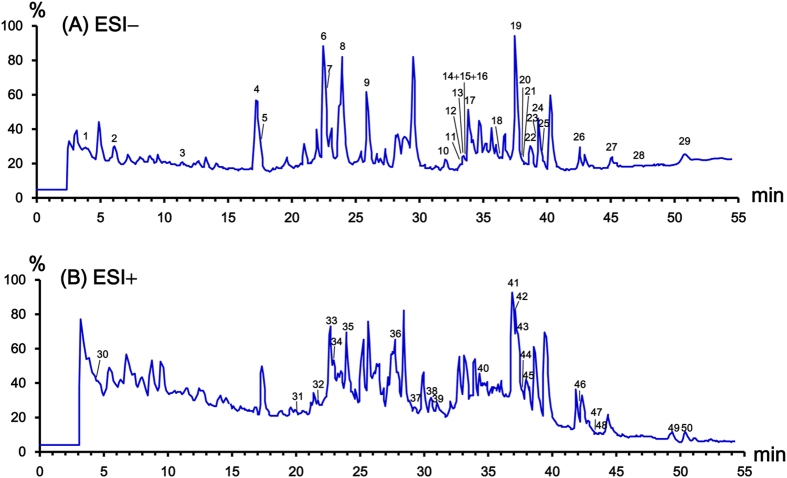
UPLC-QTOF/MS spectra of K-601. (**A**) Spectra in negative ion mode. (**B**) Spectra in positive ion mode.

Assignment of peaks in chemical profile of herbal products is challenging, since most of reference compounds are unavailable for structural confirmation. A diagnostic-ion screening strategy was proposed. Briefly, the diagnostic ions corresponding to a mother skeleton obtained from reference compounds are used to screen the same type of compounds^5^,^21^. Then, the molecular ions of screened peaks were calculated using both negative and positive ion modes. Next, the accurate molecular formula of each peak obtained was applied to screening for a hit against various chemical databases. A most possible structure that contains such a substructure and substituent groups can be determined from these candidates by comparison of characteristic product ions and fragmentation pathways. Some peaks were further confirmed using reference compounds. With the diagnostic ions strategy, 50 compounds were identified in K-601 sample by UPLC-QTOF/MS. The total ion chromatograms (TICs) of the extract in negative and positive ion modes were presented (Fig. 3A,B). The retention times, MS data and peak height abundance of the characterized compounds were summarized (Table 1). The herbal sources for each peak were also included. All the structures of the compounds identified in K-601 were summarized in Supplementary Figure S1.

### Identification of herbal constituents and their metabolites in human plasma

The detection and identification of chemical compositions in biosamples including prototype compounds and metabolites is a crucial step to uncover the pharmacologically active substances of herbal medicines^22^. Biological metabolic networks of complex mixtures were characterized by procedures of (a) mass data collection, (b) endogenous interference subtraction, (c) matching the mass differences of pseudomolecular ions between the metabolites and parent compounds based on typical metabolic pathways, (d) confirming the absorbed compounds by MS/MS product ions.

A total of 15 prototype compounds and 17 metabolites were identified. These identified metabolites were selected based on their peak abundances and intensities. Their MS data and peak height abundances of the characterized compounds were summarized (Tables 2 and 3).

### Pharmacokinetics

**Figure 4:**
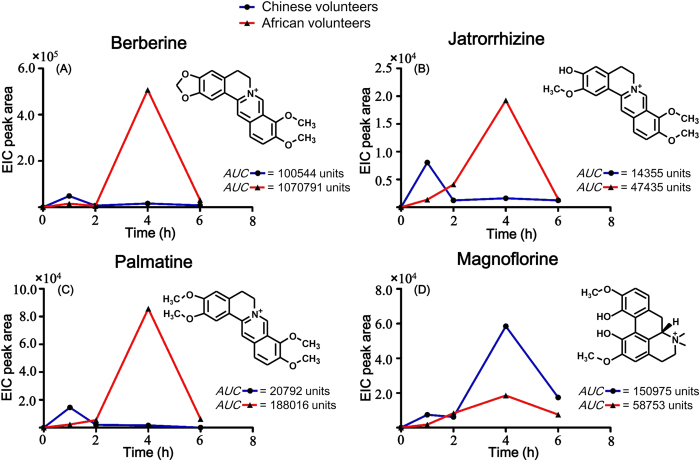
Peak area-time curves of the major prototype compounds in K-601 for Chinese and African volunteers. (**A**) Berberine. (**B**) Jatrorrhizine. (**C**) Palmatine. (**D**) Magnoflorine.

The pharmacokinetics of the four major compounds, i.e., berberine, jatrorrhizine, palmatine, and magnoflorine were determined. The peak area against time for the subjects is presented (Fig. 4). Their average areas under the concentration curves, *AUC* in the plasma after a single oral administration of 40 mL of K-601 was also presented (Fig. 4).

### Effect of intestinal flora on the metabolism and biotransformation of K-601

This experiment was done to assess the influence of intestinal flora on the metabolism and biotransformation of K-601 shown in Supplementary Figure S2. Aside the metabolism by the liver, the intestinal microbiota could play an initial metabolic role on drugs before absorption. Using the flora from the human fecal specimen, a total of 28 metabolites were tentatively identified (Table 4).

## Discussion

Herbal preparations have gained growing popularity worldwide. Because of chemical complexity, little is known about their pharmacokinetics in humans. K-601 is a hospital-prepared herbal formulation extensively used for treatment of influenza in China. In this work, we characterized the chemical constituents in K-601, identified the absorbed compounds and determined their pharmacokinetics in 6 Chinese and African volunteers by UPLC-Q/TOF-MS.

The quality of the K-601 formulation was evaluated by lot-to-lot consistency. Chromatograms from nine batches of the formulation were assessed. The results showed a high consistency among various batches. For the qualitative determination of K-601, we applied the diagnostic-ion screening strategy^5^,^21^,^22^. The rationale behind this method is that, since compounds in the formulation belong to one of several families such as flavonoid, glycosides, alkaloid, etc., each one has a characteristic carbon skeleton. Homologous compounds share the same structural units, thus a common fragmentation pathway, specific to that family of compounds. Using the diagnostic fragmentalion screening strategy, 50 compounds were identified in the formulation. Some of these compounds were further confirmed using available reference compounds.

Pharmacochemistry is based on the premise that only the absorbed components of a formulation could exert a therapeutic effect^23^,^24^,^25^. This includes both prototype compounds as well as metabolites. The prototype compounds with high and moderate peak abundances were selected for further pharmacokinetic studies. These were determined as berberine, jatrorrhizine, palmatine and magnoflorine. The rest of the prototype compounds identified in the plasma gave low peak abundances per our criteria. Interestingly, alkaloids were present about 100-fold higher than other compounds.

A total of 17 metabolites were identified. The major metabolic pathways of the detected metabolites were glucuronidation, sulfation, methylation, demethylation, and reduction. These pathways can be traced to the two phases of metabolism, phase I and phase II. Phase I metabolism usually converts a parent drug to more polar (water soluble) active metabolites by unmasking or inserting a polar functional group such as −OH, −SH, −NH_2_ etc. Oxidation and hydrolysis constitute some examples of phase I metabolism. Glucuronidation, acetylation and sulphation reactions are examples of phase II metabolism. These ‘conjugation reactions’ also increase the water solubility of a drug molecule with a polar moiety such as acetate, glucuronide or sulphate.

The pharmacokinetics of the four major prototype compounds were different in the African and Chinese volunteers. The *AUC* values for the African volunteers were higher than that of the Chinese with respect to the three benzylisoquinoline alkaloids (berberine, jatrorrhizine, and palmatine). Magnoflorine, one of the aporphine alkaloids, performed better in the Chinese volunteers than in the Africans. The time taken for these prototype compounds to reach maximum concentration (*T*_*max*_) in the blood was another major difference detected. The *T*_*max*_ for berberine, jatrorrhizine, and palmatine was 4 hours in the African volunteers corresponding to the results in rat model^26^. While for the Chinese volunteers, *T*_*max*_ was observed at 1 hour. These results go to proof that racial and structure differences play crucial roles in the pharmacokinetics of drugs, and thus influences dosing. Three possible explanations could be given for the difference: (1) The African volunteers absorb the drug slower and better or metabolize the drug slower than the Chinese volunteers. (2) The drug is poorly absorbed by the Chinese volunteers or they quickly metabolize the drug. (3) Structures may play a vital role in their ability to be absorbed or metabolized.

We investigated the possible role of intestinal microbiota in the biotransformation and metabolism of the components of K-601. This was done using flora from feces of an adult male African. The results of the study revealed after 48 hours of anaerobically incubating K-601 with the intestinal flora, that biotransformation and metabolism took place. These metabolites were found to be from the parent compounds of gallic acid, secologanoside, 4-*O*-caffeoylquinic acid and its isomers, emodin, lotusine, palmatine, berberine and baicalin. The metabolic pathways included hydroxylation, methylation, sulfation, acetylation, demethylation and reduction. These metabolites were found in high, moderate and low concentrations. Metabolites of emodin, lotusine, palmatine, berberine were detected in high concentrations. Glucuronide conjugates were conspicuously missing as metabolites. These might be transformed after liver metabolism. It should be noted that since the intestinal microbiota contains trillions of bacteria, representing species and subspecies^27^, the environment of these bacteria is not constant. Variable population of intestinal flora may lead to diverse metabolic results depending on the host conditions such as diet, health and even stress^28^. Several factors could affect the metabolism of herbal medicines. Due to competitive absorption, metabolism and exposure of different concentrations of the constituents *in vivo* and *in vitro*, the metabolic profiles of K-601 may differ^29^.

The following are the limitations of this study: (1) The sample size for the study (6 persons) was small and not adequate. This was because we could not get more than the 6 persons to volunteer for the study since the study was conducted in China where the African population is very small. (2) Though the subjects fasted throughout the period of study, six hours might not be adequate to comprehensively determine the pharmacokinetics of the all components.

## Methods

### Identification of chemical components of K-601

#### Sample preparation

In order to comprehensively determine the metabolites of the formulation upon ingestion, the chemical composition was first determined. 1 mL of this herbal product was dissolved in 1 mL of distilled water (purified by Milli-Q system, Millipore, USA). The resultant solution was centrifuged at 13000 rpm for 10 min and then the supernatant was transferred to a sample vial for UPLC-MS analysis.

#### UPLC-QTOF/MS analysis

Chromatographic analysis was performed on an Agilent 1290 Series (Agilent Corp., Santa Clara, CA, USA,) UPLC system equipped with a binary pump, micro degasser, an auto sampler and a thermostatically controlled column compartment. Chromatographic separation was carried out at 25 ^o^C on a Zorbax RRHD Eclipse Plus C_18_ column (2.1 × 50 mm, 1.8 μm). The mobile phase consisted of 0.1% formic acid solution (A) and ACN (B) using a gradient elution of 0–5% B at 0–6 min, 5–8% B at 6–15 min, 8–15% B at 15–20 min, 15–20% B at 20–30 min, 20–30% at 30–35 min, 30–35% at 35–45 min, 35–40% at 45–60 min. The flow rate was kept at 0.2 mL/min, and the sample volume injected was set at 5 μL. Detections were carried out by Agilent 6530 Q/TOF mass spectrometer (Agilent Corp., Santa Clara, CA, USA) equipped with an ESI interface. The parameters of operation were as follows: drying gas N_2_ flow rate, 10.0 L/min; temperature, 330 ^o^C; nebulizer, 35 psig; capillary, 3000 V; skimmer, 60 V; OCT RFV, 250 V. Each sample was analyzed in both the positive and negative modes due to the selective sensitivities to different components of the formulation-providing better information for molecular formulae and structural identification. Mass spectra were recorded across the range *m/z* 100–1000 with accurate mass measurements.

### Quality Evaluation of K-601

#### Sample preparation

The sample preparation was same as stated above (identification).

#### UPLC analyses

Chromatographic separation conditions were same as that used for UPLC-QTOF/MS. However, detection was done at the wavelength of 360 nm with DAD detector.

#### Data analyses

The chromatographic peaks were introduced into the *Similarity Evaluation System for Chromatographic Fingerprint of Traditional Chinese Medicine* (version 2004A, National Committee of Pharmacopoeia, China).

### Effect of intestinal flora on K-601

#### Human fecal sample preparation

The method used for the preparation of human fecal specimen was according to that already reported^30^. Briefly, 3 g of fresh human feces from a healthy male (31 years old, non-smoker, not on any medication especially antibiotics and fasting as of the time of sample collection) was weighed into a beaker. This was then suspended in 30 mL normal saline solution, filtered through a gauze and centrifuged at 13000 rpm for 30 min. The supernatant was filtered with gauze and the resultant filtrate was used as the intestinal flora fraction.

#### Biotransformation and metabolism of K-601 by human fecal flora

Three (3) conical flasks containing 30 mL of anaerobic physiological media labelled A, B and C were used. To flask A, was added 1 mL of K-601 and 2 mL of intestinal flora. To flask B was added only 2 mL of intestinal flora while only 1 mL of the K-601 was added to the content of flask C. The contents of these 3 flasks were anaerobically incubated at 37 ^°^C for 48 hours. The mixtures were then extracted 3 times with 50 mL ethyl acetate. The remaining mixtures were re-extracted 3 times with 50 mL *n*-butanol. The combined *n*-butanol extracts were then washed 3 times with water. Both extracts were concentrated in vacuo and then diluted to the desired volume with methanol. The ethyl acetate and *n*-butanol extracted contents were mixed and centrifuged at 13000 rpm for 10 min before injected for analysis.

### Pharmacokinetics study

#### Study subjects

A total of six healthy male volunteers, ages ranging from 22–47 years took part in this study. Three of whom are Africans and three Chinese. All volunteers avoided the intake of alcohol/alcoholic beverages, coffee/beverages containing coffee for at least 12 hours prior to the study. None was also on any medication. All volunteers also fasted for 12 hours prior to the study and throughout the study period.

Blood samples were withdrawn from subjects at the following time intervals, 0 hour (before taking medication and breakfast), 1, 2, 4, and 6 hours after taking the medication. These blood samples were taken by a qualified phlebotomist in the hospital. Each volunteer took 40 mL of same batch of the medication as a single dose. This study was approved by the Ethics Committee of the First Affiliated Hospital of Nanjing Medical University (2013-SRFA-078) and conducted under the guidelines of the Helsinki Declaration and the International Conference on Harmonization-Good Clinical Practices (ICH-GCP). Details of subjects in the pharmacokinetic studies are provided (Table 5).

### Treatment of plasma samples

All blood samples taken at each time were immediately centrifuged at 13000 rpm for 10 min, and the plasma separated and stored at −80 ^°^C until analysis. Plasma samples were thawed at 37 ^°^C before solid-phase extraction (SPE) treatment for UPLC-QTOF/MS analysis. The sample treatment procedure is schematically presented in Supplementary Figure S3.

#### Pharmacokinetics Analysis

Pharmacokinetic analyses were done using the extracted ion chromatograms (EIC) of the most abundant compounds. The peak areas of derived from the EIC were plotted against time (h).

Ethical Standards: All participants were required to give a written, informed consent. The studies was approved by the ethical committee and conducted in accordance with the Helsinki Declaration and Good Clinical Practice guidelines of ICH. 

## Additional Information

**How to cite this article**: Alolga, R. N. *et al.* Pharmacokinetics of a multicomponent herbal preparation in healthy Chinese and African volunteers. *Sci. Rep.* **5**, 12961; doi: 10.1038/srep12961 (2015).

## Supplementary Material

Supplementary Information

## Figures and Tables

**Table 1:** Compounds identified in K-601 heji in positive and negative modes.

**No.**	***t*_R_(min)**	**ESI mode**	**Fragment ions, *m/z***	**Formula**	**Identity**	**Abund. (×10^6^)**	**Peak abundance**	**Source**
1	4.265	−	169.0134, 125.0246	C_7_H_6_O_5_	Gallic acid*	16.06	High	RP
2	6.174	−	331.0670, 271.0433, 211.0235, 169.0139	C_13_H_16_O_10_	Gallic acid 3-*O*-β-D-glucopyranoside	2.93	Medium	RP
3	11.765	−	389.1051, 345.1085, 183.0641, 165.0548	C_16_H_22_O_11_	Secologanoside	16.70	High	LJ
4	17.136	−	353.0871, 191.0540	C_16_H_18_O_9_	Chlorogenic acid*	4.78	Medium	LJ
5	17.36	−	179.0331, 135.043	C_9_H_8_O_4_	Caffeic acid*	3.17	Medium	LJ
6	22.593	−	353.0869, 191.0533, 179.0316, 173.0424, 161.0187, 135.0421	C_16_H_18_O_9_	4-*O*-caffeoylquinic acid	22.43	High	LJ
7	22.999	−	353.0863, 191.0531, 179.0316, 173.0324	C_16_H_18_O_9_	5-*O*-caffeoylquinic acid	21.06	High	LJ
8	24.536	−	357.1197, 195.0671, 151.0761, 125.0251	C_16_H_22_O_9_	Sweroside	9.72	Medium	LJ
9	26.053	−	367.1028, 191.0534	C_17_H_20_O_9_	Caffeoyl-CH_2_-*O*-quinic acid	8.36	Medium	LJ
10	32.45	−	269.0439, 240.0408, 211.0400	C_15_H_10_O_5_	Aloe-emodin*	0.21	Low	RP
11	33.286	−	609.1475, 301.0325	C_27_H_30_O_16_	Rutin*	0.53	Low	SC
12	33.455	−	445.0756, 283.0241, 239.0326,	C_21_H_18_O_11_	Rhein-8-*O*-β-D-glucopyranoside	0.04	Low	RP
13	33.658	−	447.0897, 285.0396	C_21_H_20_O_11_	Luteolin-7-*O*-β-D-glucoside	0.75	Low	RP
14	33.692	−	463.0895, 301.0340, 271.0291	C_21_H_20_O_12_	Hyperoside	0.42	Low	LJ
15	33.861	−	461.0737, 285.0339	C_21_H_18_O_12_	Scutellarin	0.47	Low	SC
16	33.937	−	593.1519, 447.0962, 285.0401	C_27_H_30_O_15_	Lonicerin*	0.52	Low	LJ
17	34.756	−	515.1189, 353.0873, 191.0551, 179.0346	C_25_H_24_O_12_	1,5-*O*-dicaffeoylquinic acid or 4,5-*O*-dicaffeoylquinic acid or 3,5-*O*-dicaffeoylquinic acid	0.05	Low	LJ
18	36.809	−	269.0476, 167.0507	C_15_H_10_O_5_	Baicalein*	0.12	Low	SC
19	37.817	−	269.0456, 240.0408, 211.0400, 167.0518	C_15_H_10_O_5_	Emodin*	12.33	High	RP
20	38.152	−	269.0452, 225.0539, 197.0564, 183.0448	C_15_H_10_O_5_	Apeginin	0.24	Low	LJ
21	38.701	−	253.0488, 225.0556	C_15_H_10_O_4_	Chrysophanol	0.22	Low	RP
22	39.326	−	253.0504, 225.0557	C_15_H_10_O_4_	Chrysin	0.31	Low	RP
23	39.410	−	431.0987, 395.1032, 311.0588, 269.0468	C_21_H_20_O_10_	Aloe-emodin-8-*O*-glucoside	0.40	Low	RP
24	40.027	−	459.0952, 283.0612, 268.0376	C_22_H_20_O_11_	Wogonoside*	16.97	High	SC
25	40.263	−	283.0605, 268.0363, 240.0372	C_16_H_12_O_5_	Physcion	0.72	Low	RP
26	43.895	−	269.0463, 241.0521, 225.0543	C_15_H_10_O_5_	Norwogonin	0.26	Low	SC
27	45.416	−	313.7373, 229.2099, 211.1348, 171.1012	C_18_H_34_O_5_	Sanleng acid	2.90	Medium	RP
28	47.544	−	297.0409, 253.0501, 225.0517	C_16_H_10_O_6_	6-Methyl-rhein	0.17	Low	RP
29	52.055	−	283.0260, 239.0343, 211.0411, 183.0413	C_15_H_8_O_6_	Rhein*	0.28	Low	RP
30	4.076	+	180.1286, 121.0535	C_11_H_18_NO	Candicine	4.02	Medium	PC
31	20.496	+	314.1716, 269.1167, 237.0903, 192.1009, 143.0486	C_19_H_23_NO_3_	Lotusine	24.34	High	PC
32	22.430	+	342.1687, 192.1007	C_20_H_24_NO_4_	Phellodendrine*	25.40	High	SC
33	23.461	+	344.1854, 299.1283, 207.0766, 175.0748, 137.0590	C_20_H_26_NO_4_	Tembetarine	20.21	High	PC
34	23.498	+	342.1730, 297.1121, 265.0859	C_20_H_23_NO_4_	Magnoflorine*	25.39	High	PC
35	24.120	+	342.1684, 297.1102, 265.0852	C_20_H_23_NO_4_	Tetrahydrojatrorrhizine	25.32	High	PC
36	28.613	+	356.1853, 311.1271, 279.1016	C_21_H_25_NO_4_	Menisperine	22.31	High	PC
37	30.26	+	312.1591, 297.1361, 267.1191, 252.1016	C_19_H_21_NO_3_	Veticuline	0.32	Low	PC
38	31.966	+	356.1854, 192.1017	C_21_H_25_NO_4_	Tetrahydropalmatine	24.19	High	PC
39	31.975	+	356.1855, 297.0821, 192.1017, 177.0780, 148.0752	C_21_H_26_NO_4_	N-Methyltetrahydrocolumbamine	23.19	High	PC
40	35.193	+	338.1377, 322.1072, 308.0908, 294.1118, 280.0951	C_20_H_20_NO_4_	Jatrorrhizine*	2.03	Medium	PC
41	37.544	+	352.1534, 336.1228, 322.1074, 308.1277, 294.1121	C_21_H_22_NO_4_	Palmatine*	25.38	High	PC
42	37.722	+	336.1233, 334.1071, 306.0764, 292.0968	C_20_H_18_NO_4_	Berberine*	23.26	High	PC
43	37.783	+	445.0772, 271.0608	C_21_H_18_O_11_	Baicalin*	24.40	High	SC
44	38.119	+	320.0911, 292.0962	C_20_H_18_NO_4_	Epiberberine	0.06	Low	PC
45	38.600	+	263.0820	C_16_H_10_N_2_O_2_	Indigotin	0.25	Low	II
46	42.955	+	437.3389, 409.3480, 366.0660	C_26_H_30_O_7_	Obacunone	0.12	Low	PC
47	44.121	+	329.2210, 316.0589, 301.0377, 287.0624, 273.0374	C_17_H_14_O_7_	Iristectorigenin A or Iristectorigenin B	1.73	Medium	LJ
48	44.121	+	329.2210, 316.0589, 301.0377, 287.0624, 273.0374	C_17_H_14_O_7_	Iristectorigenin A or Iristectorigenin B	1.73	Medium	LJ
49	50.202	+	453.1858, 425.1962, 367.1909	C_26_H_30_O_8_	Obaculactone	2.33	Medium	PC
50	51.52	+	285.0760, 270.0522	C_16_H_12_O_5_	Wogonin*	2.80	Medium	SC

The relative abundance of the compounds measured by the peak height in the **EIC > 10.00 × 10**^**6**^, defined as major constituent, thus high level**; (1.00–10.00) × 10**^**6**^, defined as minor constituent, meaning moderate level; **<1.00 × 10**^**6**^ defined as trace constituent, hence present in low levels.

**PC**: *Phellodendron chinense* Schneid **LJ**: *Lonicera japonica* Thunb **II**: *Isatis indigotica* Fort **SC**: *Scutellaria baicalensis* Geo **RP**: *Rheum palmatum* L.

^*^Authenticated with reference standards.

**Table 2:** Prototype compounds identified in the plasma of study subjects.

**No.**	**t_R_(min)**	**ESI mode**	***m/z***	**Formula**	**Identity**	**Abund. (×10^4^)**	**Peak abundance**
P1	22.481	+	342.1692	C_20_H_23_NO_4_	Magnoflorine	6.52	Moderate
P2	24.166	+	356.1854	C_21_H_25_NO_4_	Tetrahydropalmatine	0.03	Low
P3	35.154	+	338.1375	C_20_H_20_NO_4_	Jatrorrhizine	3.53	Moderate
P4	36.507	+	352.1534	C_21_H_22_NO_4_	Palmatine	12.63	High
P5	36.738	+	336.1229	C_20_H_18_NO_4_	Berberine	50.35	High
P6	11.756	−	389.1058	C_16_H_22_O_11_	Secologanoside	0.02	Low
P7	15.863	−	353.0843	C_16_H_18_O_9_	Chlorogenic acid	0.03	Low
P8	22.539	−	353.0854	C_16_H_18_O_9_	4-*O*-caffeoylquinic acid	0.04	Low
P9	27.035	−	367.1028	C_17_H_20_O_9_	Caffeoyl-CH_2_-*O*-quinic acid	0.03	Low
P10	33.654	−	463.0890	C_21_H_20_O_12_	Hyperoside	0.05	Low
P11	34.787	−	515.1155	C_25_H_24_O_12_	1,5-*O*-dicaffeoylquinic acid or 4,5-*O*-dicaffeoylquinic acid or 3,5-*O*-dicaffeoylquinic acid	0.06	Low
P12	36.820	−	269.0439	C_15_H_10_O_5_	Emodin	0.04	Low
P13	40.059	−	459.0915	C_22_H_20_O_11_	Wogonoside	0.42	Low
P14	52.048	−	283.0260	C_15_H_8_O_6_	Rhein	0.62	Low
P15	47.536	−	297.0411	C_16_H_10_O_6_	6-Methyl-rhein	0.06	Low

The relative abundance of the compounds measured by peak height in the **EIC > 1.00 × 10**^**4**^, defined as major constituent, thus high-level**: (0.10–1.00) × 10**^**4**^ as minor constituent, meaning moderate level: **<0.10 × 10**^**4**^ as trace constituent, thus low-level.

**Table 3:** Metabolites identified in human plasma after administration of K-601.

**No.**	***t*_R_(min)**	**ESI mode**	***m/z***	**Metabolic pathway**	**Formula**	**Parent compound**	**Abund. (×10^4^)**	**Peak abundance**	
M1	7.600	−	250.9807	Reduction+Sulfation	C_7_H_8_O_8_S	Gallic acid	0.02	Low	
M2	9.927	−	183.0284	Methylation	C_8_H_8_O_5_	Gallic acid	0.21	Moderate	
M3	9.229	−	155.0713	Reduction	C_7_H_6_O_4_	Secologanoside	0.04	Low	
M4	20.380	−	393.1383	Reduction	C_16_H_24_O_11_	Secologanoside	0.03	Low	
M5	6.999	−	407.1549	Reduction+methylation	C_17_H_26_O_11_	Secologanoside	0.03	Low	
M6	31.328	−	255.0639	Reduction	C_15_H_10_O_4_	Emodin	0.05	Low	
M7	34.458	−	445.0760	Glucuronidation	C_21_H_18_O_11_	Emodin	0.50	Moderate	
M8	26.855	−	447.0938	Reduction+glucuronidation	C_21_H_20_O_11_	Emodin	0.03	Low	
M9	38.918	−	283.0604	Methylation	C_16_H_12_O_5_	Emodin	0.03	Low	
M10	43.378	−	255.0286	Demethylation	C_14_H_8_O_5_	Emodin	0.02	Low	
M11	31.429	+	447.0923	Glucuronidation	C_21_H_18_O_11_	Emodin	0.50	Moderate	
M12	9.533	+	358.2074	Reduction+methylation	C_21_H_28_NO_4_	Phellodendrine	2.00	High	
M13	32.037	+	352.1536	Reduction+methylation	C_21_H_22_NO_4_	Berberine	1.50	High	
M14	23.522	+	463.0863	Hydroxylation	C_21_H_18_O_12_	Baicalin	0.05	Low	
M15	34.470	+	431.0965	Reduction	C_21_H_18_O_10_	Baicalin	0.06	Low	
M16	26.361	+	449.1069	Reduction	C_21_H_20_O_11_	Baicalin	0.03	Low	
M17	35.281	+	461.1084	Methylation	C_22_H_20_O_11_	Baicalin	1.20	High	

The relative abundance of the compounds measured by peak height in the **EIC > 1.00 × 10**^**4**^, defined as major constituent, thus high-level**: (0.10–1.00) × 10**^**4**^ as minor constituent, meaning moderate level: **<0.10 × 10**^**4**^ as trace constituent, thus low-level.

**Table 4:** Metabolites identified in the human intestinal transformation of K-601.

**No.**	***t*_R_(min)**	**ESI mode**	***m/z***	**Metabolic pathway**	**Formula**	**Parent compound**	**Abund. (×10^4^)**	**Peak abundance**
T1	2.187	+	185.0565	Hydroxylation	C_7_H_6_O_6_	Gallic acid	0.05	Low
T2	1.680	+	199.0260	Hydroxylation+methylation	C_8_H_8_O_6_	Gallic acid	0.10	Low
T3	5.633	+	153.0203	Reduction	C_7_H_6_O_4_	Gallic acid	1.80	High
T4	7.600	+	211.0247	Sulfation	C_9_H_8_O_6_	Gallic acid	0.13	Low
T5	1.274	+	405.1042	Hydroxylation	C_16_H_22_O_12_	Secologanoside	0.04	Low
T6	22.458	+	373.1153	2×Hydroxylation	C_16_H_22_O_13_	Secologanoside	0.03	Low
T7	21.849	+	431.1184	Acetylation	C_18_H_24_O_12_	Secologanoside	0.02	Low
T8	21.951	+	403.1252	Methylation	C_17_H_24_O_11_	Secologanoside	4.00	High
T9	23.268	+	369.1185	Reduction+methylation	C_17_H_22_O_9_	4-*O*-caffeoylquinic acid	0.02	Low
T10	23.573	+	367.1035	Methylation	C_17_H_20_O_9_	4-*O*-caffeoylquinic acid	6.00	High
T11	24.282	+	367.1039	Methylation	C_17_H_20_O_9_	5-*O*-caffeoylquinic acid	5.50	High
T12	36.343	+	285.0408	Hydroxylation	C_15_H_10_O_6_	Emodin	3.00	High
T13	42.323	+	299.0562	Hydroxylation+methylation	C_16_H_12_O_6_	Emodin	5.00	High
T14	51.952	+	253.0306	Reduction	C_15_H_10_O_4_	Emodin	15.00	High
T15	38.269	+	301.0356	2×Hydroxylation	C_15_H_10_O_7_	Emodin	4.00	High
T16	51.040	+	283.0617	Methylation	C_16_H_12_O_5_	Emodin	30.00	High
T17	24.078	+	344.1851	Hydroxylation+methylation	C_20_H_25_NO_4_	Lotusine	7.50	High
T18	28.944	+	356.1849	Acetylation	C_21_H_25_NO_4_	Lotusine	17.00	High
T19	21.949	+	328.1914	Methylation	C_20_H_25_NO_3_	Lotusine	4.00	High
T20	23.368	+	300.1583	Demethylation	C_18_H_21_NO_3_	Lotusine	2.00	High
T21	44.760	+	368.1867	Reduction+methylation	C_22_H_26_NO_4_	Palmatine	1.50	High
T22	35.432	+	338.1386	Demethylation	C_20_H_20_NO_4_	Palmatine	10.00	High
T23	33.303	+	352.1183	Hydroxylation	C_20_H_18_NO_5_	Berberine	0.70	Moderate
T24	20.124	+	368.1157	2×Hydroxylation	C_20_H_18_NO_6_	Berberine	3.00	High
T25	35.534	+	338.1386	Reduction	C_20_H_20_NO_4_	Berberine	9.00	High
T26	37.055	+	352.1546	Reduction+methylation	C_21_H_22_NO_4_	Berberine	10.00	High
T27	35.533	−	475.0877	Hydroxylation+methylation	C_22_H_20_O_12_	Baicalin	0.02	Low
T28	36.141	−	429.0832	Reduction	C_21_H_18_O_10_	Baicalin	1.00	Moderate

The relative abundance of the compounds measured by peak height in the **EIC > 1.00 × 10**^**4**^, defined as major constituent, thus high-level**: (0.10–1.00) × 10**^**4**^ as minor constituent, meaning moderate level: **<0.10 × 10**^**4**^ as trace constituent, thus low-level.

**Table 5:** Details of subjects in the pharmacokinetic studies.

**Age**	**BMI(kg/m^2^)**	**Country of origin**
31	25.1	Ghana
31	25.7	Zambia
47	24.8	Nigeria
22	23.7	China
23	21.9	China
23	20.3	China
