# Supplementary material for: Pharmacokinetics of a multicomponent herbal preparation in healthy Chinese and African volunteers
Source: Sci Rep. 2015 Aug 13;5:12961. doi: 10.1038/srep12961 (PMC4534804; doi:10.1038/srep12961)

**Supplementary Information**

**Pharmacokinetics of a multicomponent herbal preparation in healthy Chinese and African volunteers**

Raphael N Alolga^1,†^, Yong Fan^1,†^, Gang Zhang^2,†^, Jin Li^1^, Yi-Jing Zhao^1^, Jimmy Lelu Kakila^1^,Yan Chen^3^,*, Ping Li^1^,*, Lian-Wen Qi ^1^,*

*^1^State Key Laboratory of Natural Medicines, China Pharmaceutical University, No.24 Tongjia Lane, Nanjing210009, China;*

*^2^Nanjing Children’s Hospital Affiliated to Nanjing Medical University,* *No.72, Guangzhou Road, Nanjing 210029, China;*

*^3^Department of Emergency Center, the First Affiliated Hospital of Nanjing Medical University, No. 300 Guangzhou Road, Nanjing 210029, China.*

^†^The first three authors contributed equally to this work.

*Corresponding authors. Tel: +86 25 86185231; Fax: +86 25 83271379.

E-mail addresses: Qilw@cpu.edu.cn (L.-W. Qi), [Liping2004@126.com](mailto:Liping2004@126.com) (P. Li), [Chenyandoc@163.com](mailto:Chenyandoc@163.com) (Y. Chen).

**Supplementary Table S1** Similarities of 9 batches of K-601

| Batch number of sample | Similarity (cosine ratio) |
| --- | --- |
| 150201 | 0.993 |
| 140623-4 | 0.989 |
| 140207-4 | 0.981 |
| 140217-4 | 0.991 |
| 140126-1 | 0.987 |
| 140212-2 | 0.984 |
| 140224-4 | 0.992 |
| 121226-2 | 0.991 |
| 140303-1 | 0.984 |

**Supplementary Figure S1** Structures of compounds identified in K-601.

**1**

Gallic acid

**3**

**4**

**5**

**6**

Gallic acid 3-O-β-D-glucopyranoside

**2**

**7**

5-O-caffeoylquinic acid

**8**

**9**

**13**

**14**

**15**

**16**

Secologanoside

Chlorogenic acid

Caffeic acid

4-O-caffeoylquinic acid

**10**

**11**

Sweroside

Caffeoyl-CH_2_-O-quinic acid

**12**

Rutin

Aloe-emodin

Lonicerin

Rhein-8-O-β-D-glucopyranoside

Hyperoside

Luteolin-7-O-β-D-glucoside

Scutellarin

**17**

**17**

**17**

**18**

**19**

**20**

1, 5-O-dicaffeoylquinic acid

4, 5-O-dicaffeoylquinic acid

3, 5-O-dicaffeoylquinic acid

Baicalein

Emodin

Apeginin

**21**

**22**

**23**

**24**

**25**

**26**

**27**

Chrysophanol

Norwogonin

Chrysin

Aloe-emodin-8-O-glucoside

Wogonoside

Physcion

Sanleng acid

**28**

**29**

**30**

**31**

**32**

**33**

**34**

**35**

6-Methyl-rhein

Rhein

Candicine

Lotusine

Phellodendrine

Tembetarine

Magnoflorine

Tetrahydrojatrorrhizine

**37**

**38**

**39**

**40**

**41**

**42**

**36**

Menisperine

Veticuline

Tetrahydropalmatine

N-Methyltetrahydrocolumbamine

Jatrorrhizine

Palmatine

Berberine

**43**

**44**

**45**

**46**

**47**

**48**

**49**

**50**

Baicalin

Epiberberine

Indigotin

Obacunone

Iristectorigenin A

Iristectorigenin B

Obaculactone

Wogonin

**Supplementary Figure S2** Influence of intestinal flora on the metabolism and biotransformation of K-601

**%**

**
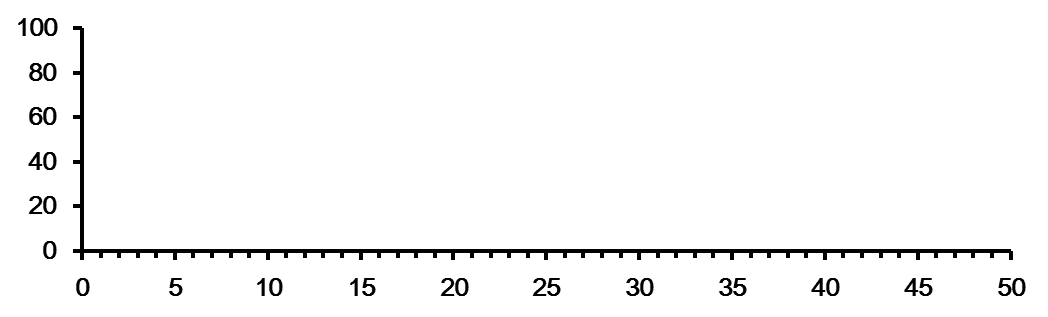
**

**(A) Bacteria + Medium**

**
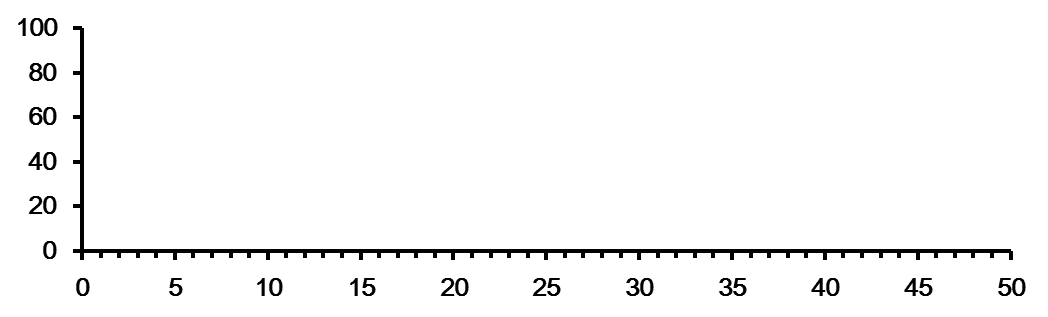

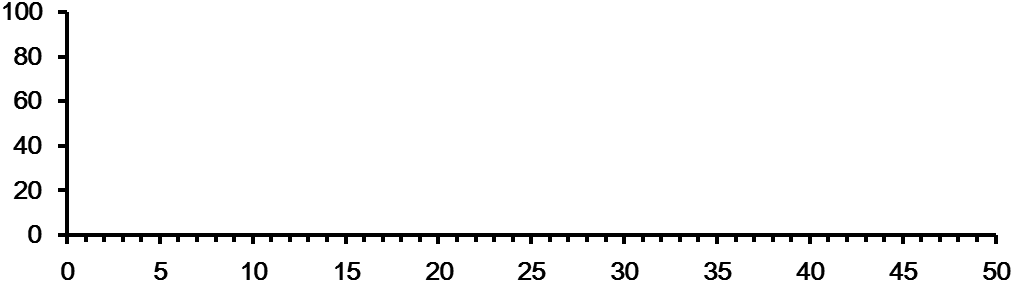
**

**min**

**min**

**min**

**%**

**%**

**(B) K-601 + Medium**

**(C) K-601 + Bacteria + Medium**

**Supplementary Figure S3** Schematic sample treatment procedure for the pharmacokinetics study of K-601.


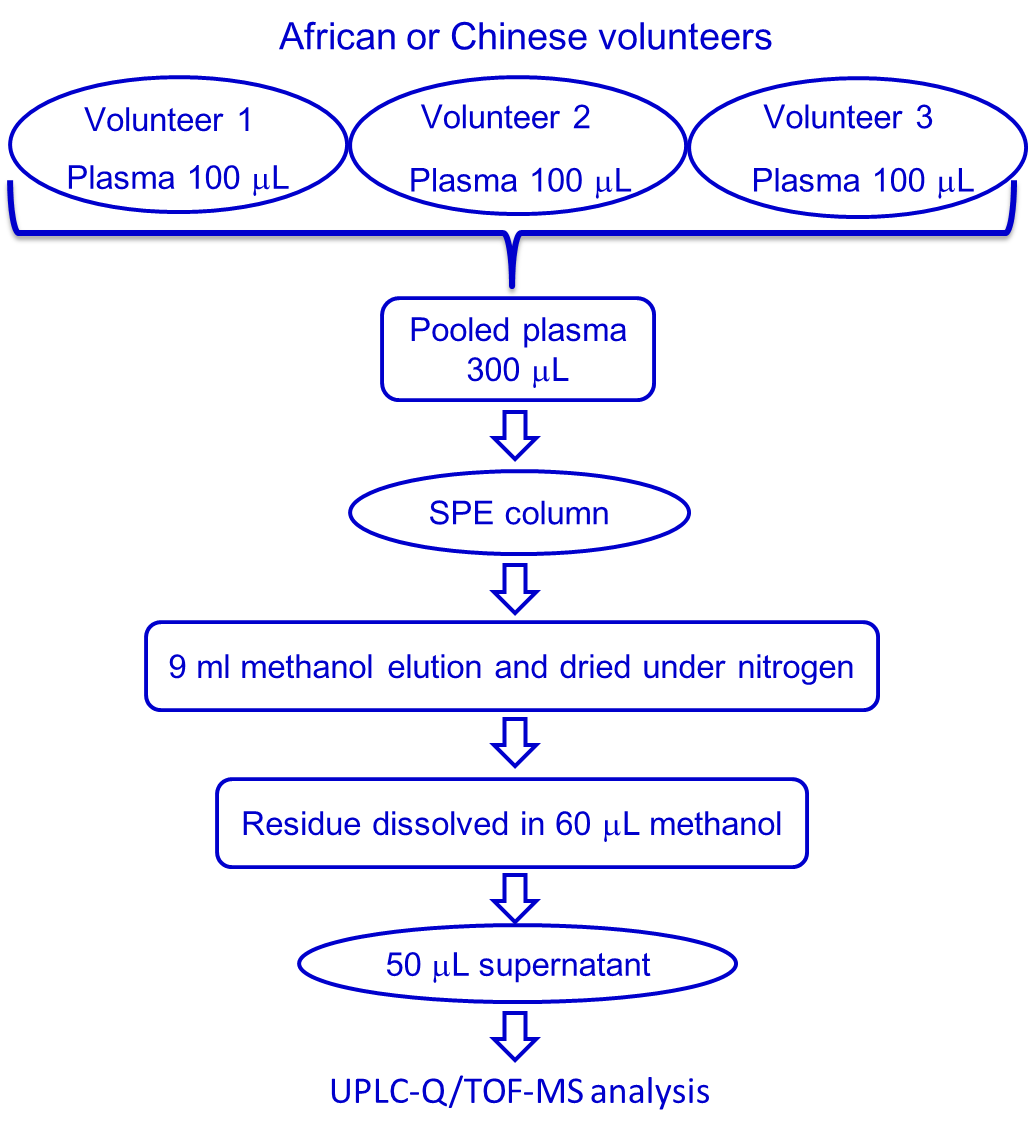

Supplement: Supplementary Information [file srep12961-s1.doc]
